# Oral administration of hyaluronic acid to improve skin conditions via a randomized double‐blind clinical test

**DOI:** 10.1111/srt.13531

**Published:** 2023-11-20

**Authors:** Yan‐Rui Gao, Rui‐Ping Wang, Lu Zhang, Yuan Fan, Jin Luan, Zhe Liu, Chao Yuan

**Affiliations:** ^1^ Department of Skin & Cosmetic Research Shanghai Skin Disease Hospital Skin Disease Hospital of Tongji University Shanghai China; ^2^ Clinical Research Center Shanghai Skin Disease Hospital Skin Disease Hospital of Tongji University Shanghai China; ^3^ Bloomage Biotech Co., Ltd Jinan Shandong China

**Keywords:** clinical benefit, hyaluronan, hyaluronic acid, oral administration, skin health

## Abstract

**Objective:**

To evaluate the impact of oral intake of Hyaluronic Acid (HA) on skin health.

**Background:**

HA, an endogenous substance in the human body, plays a key role in skin health. However, its concentration in the skin decreases significantly with age. Previous studies suggested that oral intake of HA can supplement the body's HA level, but did not reveal the effects on different age groups and skin types.

**Methods:**

A double‐blind, randomized clinical trial with 129 female participants, covering young and elderly groups and differnet skin types, was conducted to assess the efficacy of orally administered HA on skin health.

**Results:**

Oral administration of HA significantly promoted skin hydration after 2‐8 weeks among both young and elderly groups. Skin tone improvement was observed after 4‐8 weeks, while an increase in epidermal thickness was noted after 12 weeks.

**Conclusion:**

This study provides direct evidence supporting the clinical efficacy of oral intake of HA in promoting skin health.

## 1 INTRODUCTION

Hyaluronic acid (HA) is an endogenous component widely distributing in the extracellular matrix of human body, with high concentrations in connective tissues such as skin, synovial fluid, and vitreous humor.^1^ HA exhibits excellent moisturizing properties and participates in numerous physiological processes, such as wound healing, tissue repair and regeneration, inflammatory responses, embryonic development, and tumor progression.^2^, ^3^ In skin, HA plays an important role, as it not only forms hydrogen bonds with water molecules but also regulates the activity of aquaporin‐3 (APQ3), stimulates the migration and proliferation of endothelial cells, and promotes collagen synthesis.^4^, ^5^ However, with aging, the level of HA in skin decreases significantly,^6^ resulting in weakened skin tension, dryness, and wrinkles.

With the emergence of HA dietary supplements, some researchers investigated the absorption and metabolism mechanisms of orally ingested HA. Studies showed that orally ingested high‐molecular‐weight HA (HMM‐HA, MW≥100 kDa) does not undergo significant degradation in gastric fluid,^7^, ^8^ but is taken up by intestinal epithelial cells, M cells, dendritic cells, and macrophages.^9^, ^10^, ^11^, ^12^ In the lower intestinal epithelial cells, HMH‐HA interacts with TLR‐4 receptors, enabling it to be absorbed by intestinal cells.^13^ While, orally ingested low molecular weight HA (LMW‐HA, MW < 100 kDa) is mainly absorbed in the cecum and transported throughout the body via the bloodstream.^8^ HMW‐HA is primarily transported to intestinal‐associated lymphoid tissue and then transported throughout the body via the bloodstream. Scintigraphic imaging showed that intestinal‐associated lymphoid tissue took up intact HMM‐HA molecules and distributed them in connective tissues after 4 h, with orally ingested HA appearing in the joints, vertebrae, and salivary glands.^14^ In a similar study, orally ingested labeled HMM‐HA entered the bloodstream in its biologically active form and was transported to its destination without undergoing dissociation.^15^

In recent years, clinical trials were carried out to reveal that oral administration of HA can effectively improve skin condition. Kajimoto et al., Sato et al.,^16^, ^17^ and Yoshida et al.^18^ conducted randomized double‐blind, placebo‐controlled tests using LMW‐HA on patients with dry skin, and the results showed that oral administration of HA significantly increased skin hydration and improved skin dryness. Kawada et al.^19^ tested 61 patients with dry skin using HMW‐HA, and similarly observed a significant improvement in skin hydration. Further experiments by Schwartz et al.^20^ found that oral administration of HA can alleviate aging symptoms on facial skin.

However, previous studies have not further explored the effects of oral hyaluronic acid on different age groups and skin types. For example, young skin has relatively sufficient HA content, and oily skin has better moisture retention ability than dry skin. It is worth exploring whether oral administration of HA has the same positive effect on these types of skin. Therefore, a randomized, double‐blind test on 129 Shanghai healthy female panelists were carried out to investigate the clinical benefit of oral administration of HA.

## 2 METHOD

This clinical trial was approved by the ethics committee of our hospital, and the clinical trial was duly registered.

The test was conducted from September 27th 2022 to January 16th 2023, and the panelists include 61 young (18–35 years old) and 67 elderly (45–65 years old) groups, with their skin types covering oily, normal and dry. The oral administration level of HA was designed as 0 mg/day (placebo, 100% Erythritol), 100 mg/day (95% Erythritol, 5% 300 KDa HA), and 200 mg/day (90% Erythritol, 10% 300 KDa HA). The stratified randomization method was employed to divide the panelists into three different administration groups, whose population is as shown in Table 1.

**TABLE 1:** Experimental design.

Age group	Skin type	Oral administration level (mg/day)	Panelist population
Young group	Dry	0 (placebo)	6
Young group	Dry	100	8
Young group	Dry	200	7
Young group	Oily	0 (placebo)	6
Young group	Oily	100	6
Young group	Oily	200	6
Young group	Normal	0 (placebo)	8
Young group	Normal	100	7
Young group	Normal	200	7
Elderly group	Dry	0 (placebo)	9
Elderly group	Dry	100	7
Elderly group	Dry	200	6
Elderly group	Oily	0 (placebo)	6
Elderly group	Oily	100	9
Elderly group	Oily	200	7
Elderly group	Normal	0 (placebo)	7
Elderly group	Normal	100	9
Elderly group	Normal	200	8

Prior to the test, the participants were thoroughly screened by doctors to ensure that they had no skin‐related diseases, normal liver and kidney function, negative results on blood and urine tests, and negative HCG (reproductive age). Additionally, they had no known allergies to the tested product components and were not suffering from uncontrolled diseases such as diabetes, hypertension, hyperthyroidism, or hypothyroidism. The subjects did not use any dietary supplements containing collagen, hyaluronic acid, chondroitin sulfate, or any combination thereof within 3 months before the test, did not undergo any skin surgery or medical beauty procedures within 12 months before the test, and did not experience any skin trauma or sunburn within 3 months before the test.

The HA drinks used in the test were a unit dose solid beverages provided by Bloomage Biotech Co., Ltd. The HA ingredient in the formula was a food‐grade material prepared by fermentation. The panelists were required to dissolved one unit dose in 200 mL of water and consumed it every day. Also, they were required to ensure the total amount of daily water intake is 1.2∼1.4 L. During the test, the same facial cream products, that is, the same brand and lot#, were given to the panelists as their only skin care product for daily use. To be noted, this facial cream did not contain any active ingredients such as HA.

After oral administration of HA for 2, 4, 8, and 12 weeks, the CorneometerCM825 (Courage & Khazaka, Germany) was used to measure Skin hydration. Ultrasonic skin diagnostic instrument (UC22, Courage&Khazaka, German) was used to measure Skin thickness and density. Visia‐CR (Canfield Scientific, Inc. USA) was used to test Skin appearance. Chromameter CM2600D (KONICA MINOLTA, Japan) was used to detect Skin tone (ITAo). The measurements were carried out in the clinical lab with controlled temperature and humidity of 22 ± 1°C/RH 50% ± 10%. Prior to measurements, the panelists were instructed to sit quietly in the lab for at least 30 min.

Skin tone is quantified by ITA^o^ value which is derived from skin color *L* ^*^, *a* ^*^ and *b* ^*^ values, and the formula is shown as Equation 1.

(1) $${\mathrm{ITA}}^{o}=\left(180/\pi \right)*\arctan\left(\left(L^{*}-50\right)/b^{*}\right)$$

## 3 RESULTS

### 3.1 Skin hydration

**FIGURE 1:**
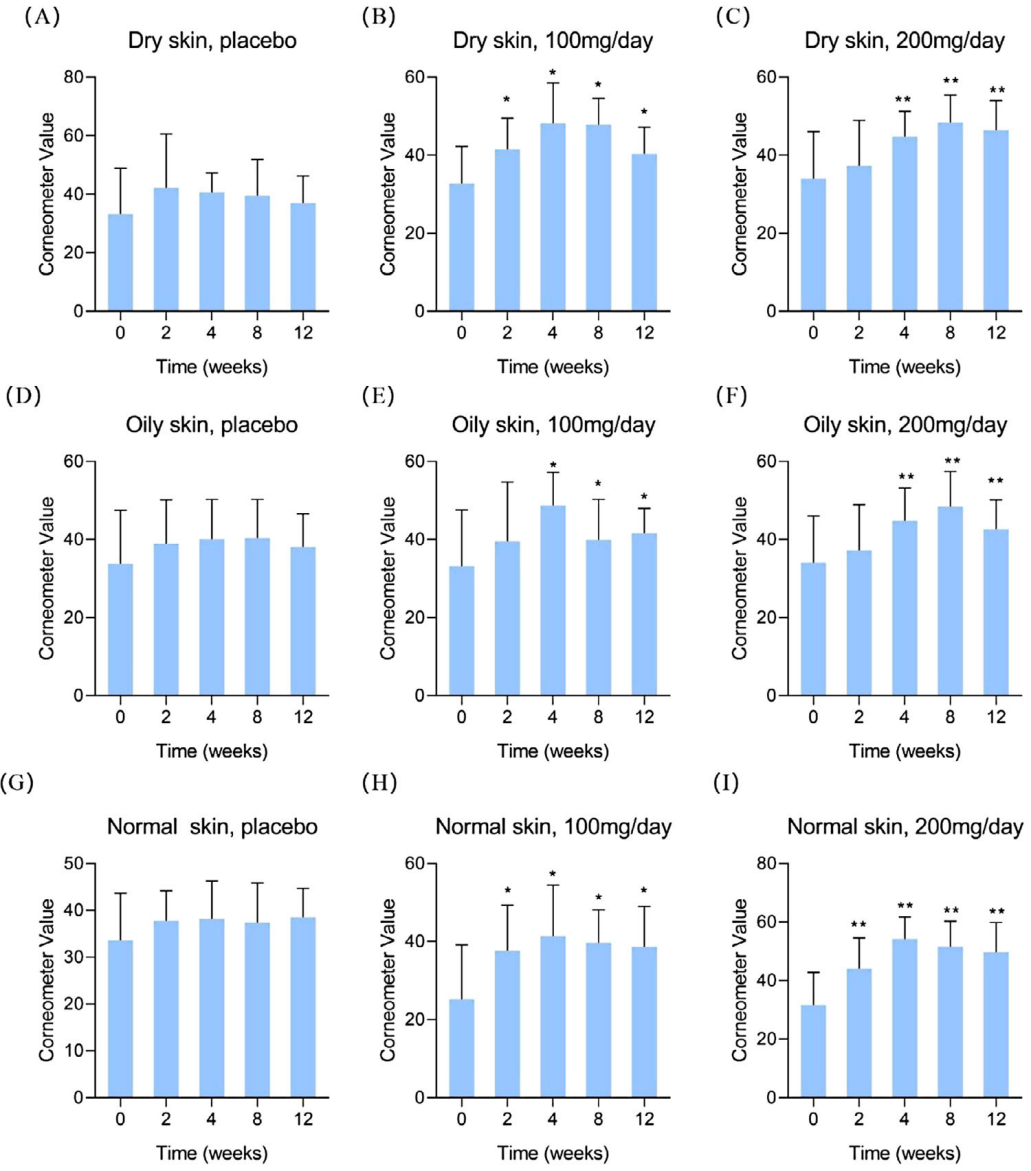
Skin hydration results of the young group by corneometer. (A) dry skin, placebo; (B) dry skin, 100 mg/day; (C) dry skin, 200 mg/day; (D) oily skin, placebo; (E) oily skin, 100 mg/day; (F) oily skin, 200 mg/day; (G) normal skin, placebo; (H) normal skin, 100 mg/day; (I) normal skin, 200 mg/day. Compared to 0 week ^*^ *p* < 0.05, ^**^ *p* < 0.01.

**FIGURE 2:**
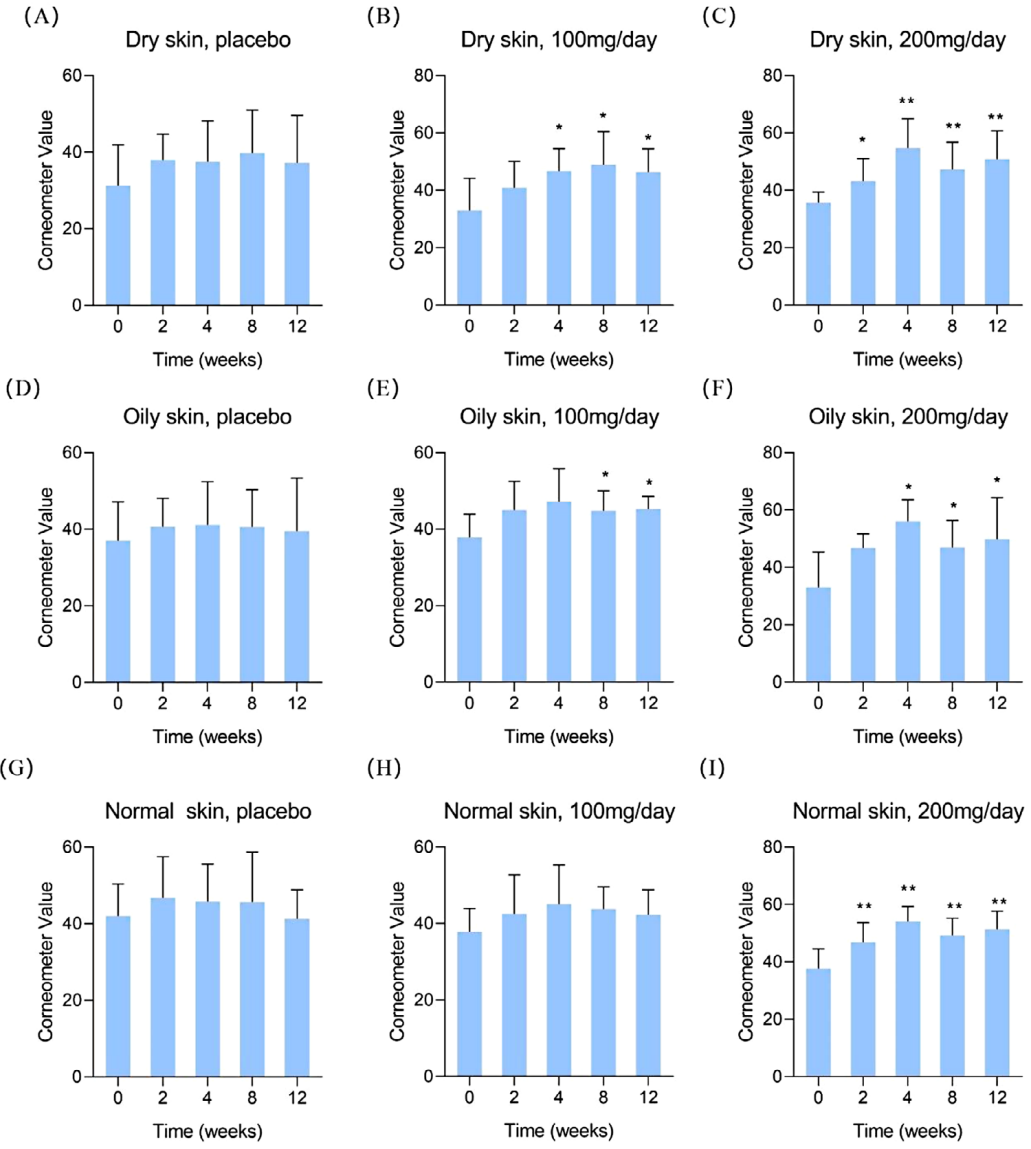
Skin hydration results of the elderly group by corneometer. (A) dry skin, placebo; (B) dry skin, 100 mg/day; (C) dry skin, 200 mg/day; (D) oily skin, placebo; (E) oily skin, 100 mg/day; (F) oily skin, 200 mg/day; (G) normal skin, placebo; (H) normal skin, 100 mg/day; (I) normal skin, 200 mg/day. Compared to 0 week ^*^ *p* < 0.05, ^**^ *p* < 0.01.

The skin hydration results of young and elderly groups were summarized in Figures 1 and 2, respectively, with separate statistics for different skin types and administration groups. The paired *t*‐test (α = 0.05) was used for data analysis, and letters were employed to indicate their differences: in the same figure, groups containing the same letter(s) do not have statistical difference, while groups containing completely different letter(s) are considered to be statistically different.

In Figure 1, the test results for dry, oily, and normal skin in the young group were summarized in (A)‐(C), (D)‐(F), and (G)‐(I), respectively. Firstly, it can be seen that no statistically change of skin hydration in the placebo group although the average values showed a slight increase trend. This trend may be contributed by two factors: (a) the basic facial cream used in the test was effective; (b) the panelists were required to drink sufficient water every day. By comparing the results in the low‐dose group, one can find the dry skin showed a hydration increase after 2 weeks of oral administration of HA, and oily and normal skin presented the significant elevation after 4 weeks. Similarly, in the high‐dose group, dry, oily and normal skin showed significant hydration improvement after 4, 8, and 12 weeks, respectively.

Moreover, the improvement of skin hydration can also be observed in the elderly group, as shown in Figure 2. For example, in the high‐dose group, dry, oily and normal skin showed statistical increases after 4, 8 and 12 weeks, respectively. However, in the low‐dose group, only dry and oily skin showed improvement, while no statistical difference was observed in normal skin, which may be due to the large standard deviation of the test results. But overall, the conclusion of oral HA to promote skin hydration is validated.

**FIGURE 3:**
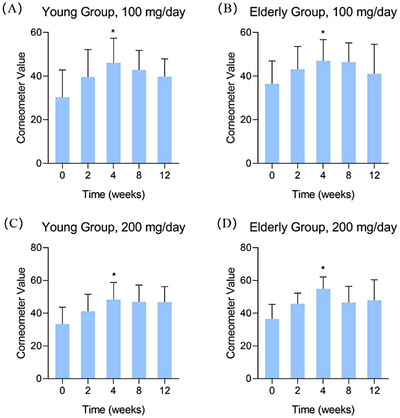
Skin hydration results with combined skin types. (A) young group, 100 mg/day; (B) elderly group, 100 mg/day; (C) young group, 200 mg/day; (D) elderly group, 200 mg/day. Compared to 0 week ^*^ *p* < 0.05.

The data of different skin types were combined for further analysis to increase the sample size. In Figure 3, it can be found that regardless of HA administration level, the improvement of skin hydration reached its peak after 4 weeks.

### 3.2 Skin tone

The results of three administration groups were summarized in Table 2, and the data of different age groups and skin types were merged. Each test point was compared with the initial value (0 week) via *t*‐test (α = 0.05), and the results revealed that there was no change in the placebo group, but a significant improvement in low‐ and high‐administration groups after 4 and 8 weeks, respectively.

**TABLE 2:** Change of ITA^o^ values at different oral hyaluronic acid administration levels.

Oral HA (mg/day)	0 week	2 weeks	4 weeks	8 weeks	12 weeks
0	38.5 (± 8.85)	39.3 (± 8.9)	39.5 (± 9.0)	39.0 (± 8.9)	39.1 (± 8.8)
100	36.7 (± 9.08)	37.5 (± 9.0)	^*^ **38.0 (± 8.7)**	37.3 (± 9.5)	36.1 (± 9.7)
200	37.4 (± 8.13)	37.8 (± 8.1)	38.2 (± 7.9)	^*^ **38.7 (± 8.4)**	37.3 (± 11.2)

Abbreviation: HA, hyaluronic acid.

* *p* < 0.05, statistical difference versus 0‐week result.

The bold values indicate statistical difference vs. 0‐week results.

### 3.3 Skin thickness and density

The results of epidermis and dermis thickness were compiled in Table 3, where the data of different age groups and skin types under identical Oral HA administration levels were merged for analysis. It was found that dermis thickness did not exhibit any statistical changes during the test period, whereas epidermis thickness showed a significant increase at the 12^th^ week with 100 mg/day administration level. The increase in epidermis thickness suggests that the skin barrier has become stronger and healthier. This is not solely contributed by the elevation of skin hydration, as the hydration level of all groups presents improvement trend.

**TABLE 3:** Change of epidermis and dermis thickness (μm) at different oral hyaluronic acid administration levels.

Skin layer	Oral HA (mg/day)	0 week	2 weeks	4 weeks	8 weeks	12 weeks
Epidermis	0	83.6 (± 14.8)	80.3 (± 9.6)	80.4 (± 10.8)	86.0 (± 15.5)	88.0 (± 18.2)
	100	80.9 (± 19.5)	79.5 (± 10.3)	77.1 (± 9.4)	81.9 (± 15.0)	^*^ **85.4 (± 14.9)**
	200	81.6 (± 8.9)	80.5 (± 10.9)	82.0 (± 10.3)	82.7 (± 18.4)	87.1 (± 16.7)
Dermis	0	1469.3 (± 285.5)	1471.8 (± 297.7)	1474.1 (± 296.2)	1501.1 (± 307.0)	1493.7 (± 272.3)
	100	1492.7 (± 239.5)	1496.7 (± 263.0)	1506.2 (± 284.7)	1511.0 (± 237.8)	1511.4 (± 289.0)
	200	1516.7 (± 280.6)	1495.1 (± 302.6)	1501.7 (± 309.7)	1498.4 (± 262.2)	1541.1 (± 278.6)

Abbreviation: HA, hyaluronic acid.

* *p* < 0.05, statistical difference versus 0‐week result.

The bold values indicate statistical difference vs. 0‐week results.

Additionally, the dermis density data were summarized in Table 4, where the data were presented as percentage values based on the intensity of ultrasound signals. After 12 weeks, the placebo group values decreased from 8.4 to 7.6, while the test groups did not demonstrate any significant changes.

**TABLE 4:** Change of dermis density (%) at different oral hyaluronic acid administration levels.

Oral HA	0 week	2 weeks	4 weeks	8 weeks	12 weeks
0	8.4 (± 2.5)	8.1 (± 2.3)	8.2 (± 2.5)	7.9 (± 2.3)	^*^ **7.6 (± 2.0)**
100	7.6 (± 1.6)	7.8 (± 2.0)	7.7 (± 2.0)	7.7 (± 1.7)	7.6 (± 1.6)
200	7.8 (± 2.2)	7.8 (± 2.4)	7.5 (± 2.1)	8.0 (± 2.2)	7.4 (± 2.0)

Abbreviation: HA, hyaluronic acid.

* *p* < 0.05, statistical difference versus 0‐week result.

The bold values indicate statistical difference vs. 0‐week results.

**FIGURE 4:**
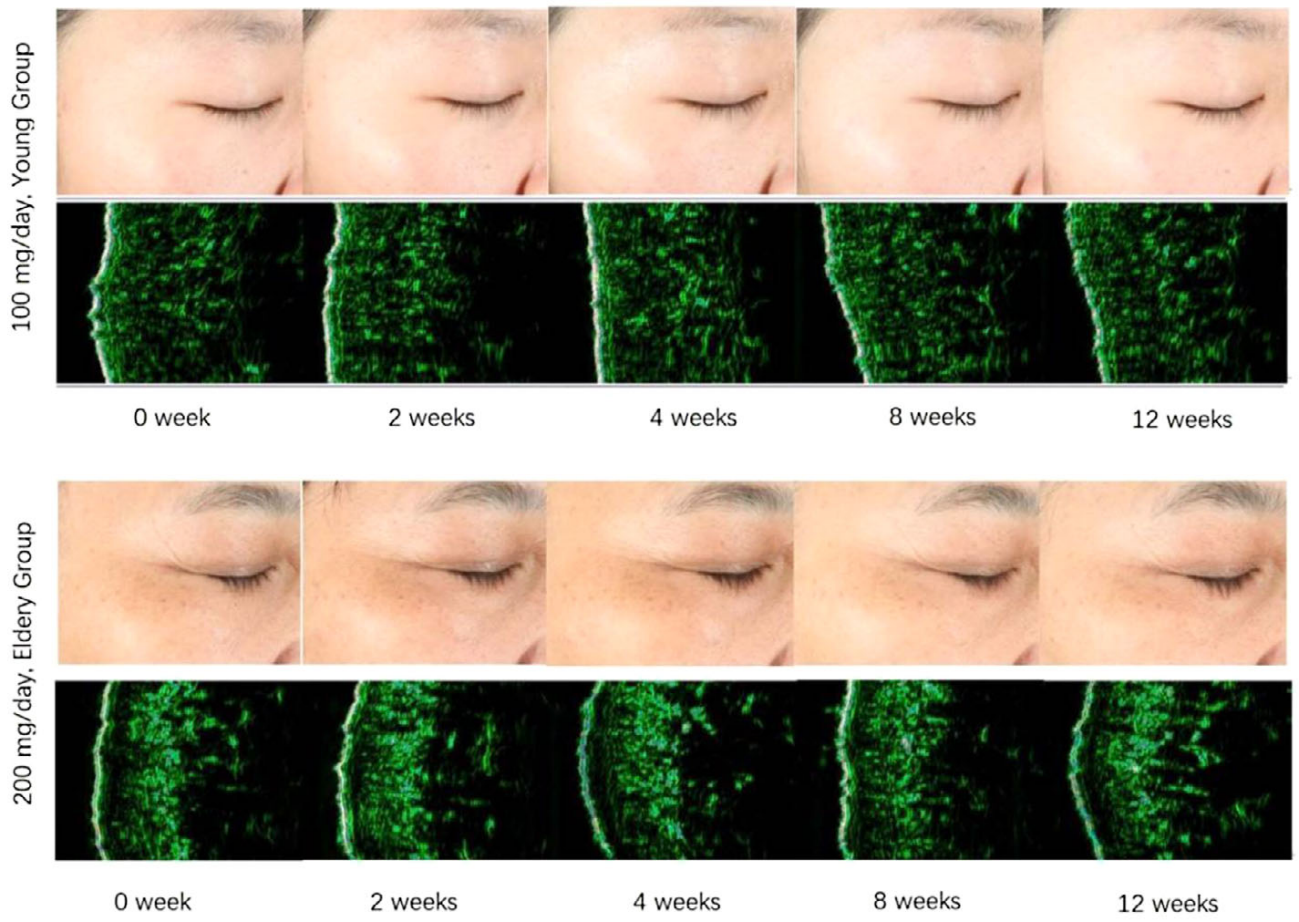
Visia and ultrasound skin diagnostic photos of subjects in the low dose and high dose groups.

One subject was selected from both low‐ and high‐administration groups, and their skin ultrasound test results were directly compared to their Visia photos as shown in Figure 4. It can be observed that the subject's fine line status significantly improved after taking HA, which is a macroscopic result of the combined effect of increased skin hydration and enhanced epidermis thickness.

## 4 DISCUSSION

Many studies have shown that HA can improve skin aging, and it also shows significant protective effects in UV‐induced photoaging models, while also showing the potential to promote skin healing.^21^, ^22^, ^23^, ^24^ It can be seen that HA plays a very important role in improving skin quality, beauty and treatment. Especially in today's society, people are more concerned about preventing aging, beauty and beauty, and the pursuit of “Whitening, firming, and youthful retention.” “Skin shines, youth is always with beauty.” Skin care and anti‐aging have been deeply embedded in People's Daily life. Based on this, we explored the effect of HA on skin improvement in different age groups.

In this study, the skin hydration results of the young group were interesting, because oral HA could further improve their skin hydration level although their skin HA levels are relatively sufficient. Then, it can be speculated that oral HA may enhance the activity of AQP3 in skin,^4^ and thus promote water transfer from dermis to epidermis.

In addition, the effect of oral administration of HA on skin hydration is not monotonic. When skin moisture rises to a certain level, it will maintain at this level or slightly decrease, but would be always higher than the initial value (0 week). Apart from its intrinsic mechanism, this phenomenon may also result from the outbreak of COVID‐19. Around the 10^th^ week of the test, a large‐scale outbreak of COVID‐19 occurred in Shanghai, and more than 63.1% of the panelist were infected, which may negatively affect their skin conditions.

Also, the negative impact of COVID‐19 can be seen in dermis density. The placebo group presented a significant lower value at the 12^th^ week versus initial. However, the test groups with oral HA maintained a stable dermis density, suggesting oral taking HA could protect skin condition.

## 5 CONCLUSION

A randomized double‐blind test on different age groups and skin types were carried out to study the clinical benefit of oral administration of HA. The results indicated that oral intake of HMW‐HA (300 KDa) at the levels of 100 and 200 mg/day can promote skin hydration after 2–8 weeks, and the effect is statistically significant for both young and elderly groups. After 4–8 weeks, it could elevate the ITA^o^ value of skin, that is, to increase skin brightness and decrease yellowness. After 12 weeks, it can promote the increase of epidermis thickness. This study provides direct evidence for the clinical efficacy of oral administration of HA.

However, there were two laminations in this study. Since the age and skin types were subdivided during analysis, the sample size of each group was relatively small, but fortunately, the entire pool was also analyzed for conclusion drawing. In addition, the study was affected by COVID‐19 pandemic, which resulted in the presence of multiple variables, especially in the middle and later stages of the test.

## CONFLICT OF INTEREST STATEMENT

The authors declare that they have no competing interests.

## ETHICS STATEMENT

This clinical trial was approved by the Ethics Committee of the Skin Disease Hospital of Tongji University (ethics approval number: 2022‐11), and the clinical trial was duly registered (registration number: ChiCTR2300073814). All methods were carried out in accordance with the Declaration of Helsinki. All patients provided written informed consent prior to enrollment in the study.

## PATIENT CONSENT FOR PUBLICATION

This study obtained the Informed consent of the patient for publication, and the researcher will protect the patient's privacy when publishing the article.

## Data Availability

The data used to support the findings of this study are available from the corresponding author upon request.
